# Initial inflammatory response of skeletal muscle to commonly used suture materials: An animal model study to evaluate muscle healing after surgical repair – histopathological perspective

**DOI:** 10.4317/medoral.18608

**Published:** 2013-03-25

**Authors:** Darpan Bhargava, P. Anantanarayanan, Geetha Prakash, B. J. Dare, Ashwini Deshpande

**Affiliations:** 1 MDS, MOMSRCPS(Glasg), PDCR, PGDHM - Assistant Professor, Department of Oral and Maxillofacial Surgeon, Peoples College of Dental Sciences & Research Center, Bhopal (M.P) India; 2 MDS, DNB, MNAMS – Professor, Department of Oral & Maxillofacial Surgery, Meenakshi Ammal Dental College & Hospital, Alappakkam Main Road, Maduravoyal, Chennai (T.N) India; 3 M.D - Professor & Head of the Department, Pathology, Meenakshi Medical College and Research Institute (MMC & RI), Kanchipuram (T.N) India; 4 MVSc, Ph.D - Director, Biomedical Research Unit & Lab Animal Center (BRULAC), Saveetha University, Chennai (T.N) India; 5 MDS, PGDHSc – Assistant Professor, Department Oral Medicine and Maxillofacial Radiology, Peoples Dental Academy, Bhanpur, Bhopal (M.P) India

## Abstract

Objectives: To evaluate initial inflammatory response of skeletal muscle to a few commonly used suture materials for muscle repair namely nylon, polydiaxonone (PDS II), plain catgut and polygalactin 910 which in turn determines the scarring of muscle and loss of function. Material and Methods: Inflammation and healing of muscle post repair was evaluated in the lateral thigh muscle (biceps femoris) of 8 adult healthy male Rattus norvegicus. The inflammatory reaction & healing of the skeletal muscle was evaluated histologically at the end of 48 hours, 1 week and 3 weeks. Results: At 48 hours post-surgery, Nylon samples showed severe inflammation followed by Catgut and Polygalactin. At 1 week post-surgery, the catgut group demonstrated increased macrophages infiltration while Nylon demonstrated persistant lymphocytic pro-inflammatory component. PDS sutures elicited minimal inflammatory response all through. Conclusion: In the present study the most desirable suture material was determined to be PDS due to its minimal tissue response and superior handling qualities. However the fact that the presence of macrophages in healing muscle enhances the repair process would be a pointer to create an environment which contains the sustained presence of macrophages to enhance optimal healing of skeletal muscle in the presence of an ideal suture material.

** Key words:**Muscle healing, muscle repair, inflammatory response to sutures, sutures, healing.

## Introduction

Healing of muscle tissue after surgical repair is influenced both by intrinsic and extrinsic factors that initiate an inflammatory response. (1,2) The intrinsic reaction is a measure of the natural immune response to injury while extrinsic factors include inflammatory response to the presence of a foreign body such as suture material at the site of repair. Therefore tissue reaction to these materials is one of the crucial factors to be considered while choosing the best suture for the surgical repair. Most surgeries performed in the maxillofacial region iatrogenically violates the muscular envelope of the face and the oral cavity. Similarly, procedures involving the jaws infringe on the muscles of mastication, which makes it the duty of the surgeon to restore these muscles to near normal status, both, anatomically and functionally. Surgeries for correction of oro-facial clefting is one such specialised area where high priority is given to muscle repair as it dictates esthetic and functional outcome and helps in restoration of important physiological activities like speech, mastication and deglutition, and also improves the quality of life of the patient. (3) In the present study we have evaluated initial inflammatory response of skeletal muscle to a few commonly used suture materials for muscle repair: nylon, polydiaxonone (PDS II), plain catgut and polygalactin 910.

## Material and Methods

Muscle repair in terms of inflammation and healing with fibrous component is evaluated in the lateral thigh muscle (biceps femoris) of 8 adult healthy male albino rats (Rattus norvegicus). Both the hind limbs of each animal were included for the study (16 sample sites). The biceps femoris was used as it had easy accessibility and adequate muscle bulk.

-Ethical Committee permission

Necessary Institutional Animal Ethics Committee (IAEC) permission was obtained. Care of animals was carried out as per the norms of “Committee for the purpose of control and supervision of experiments on animals” (CPCSEA) at Biomedical Research Unit and Lab Animal Centre (BRULAC), Chennai (India).

-Preparation of the animal sample

Common albino laboratory rats were chosen to mimic mammalian inflammatory response for the study due to its ease of availability and maintenance. All the animals were male with a weight range of 160 to 180 grams and an average age range of 3 to 4 months. All study animals were isolated from other laboratory animals one month before the commencement of the study and were fed similar diet (rat pellets) and water from a common source to standardise their nutrition and maintenance.

-Grouping of the animal sample

The sample of 8 rats was divided into four groups (Groups 1 to 4) of 2 rats each (4 surgical sites) depending on the four different suture materials used respectively. Each study group had 4 surgical sites as explained earlier. The grouping of the animal sample and the suture material used in each group is summarised in  Table 1.

**Table 1:**
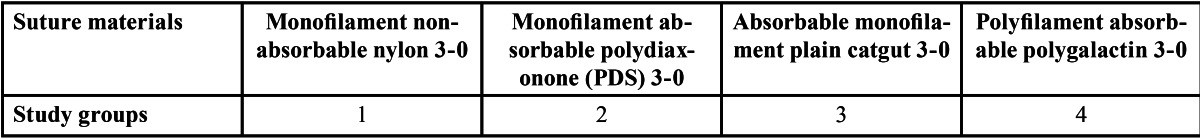
Grouping of the animal sample.

-Surgical Procedure

**Figure 1:**
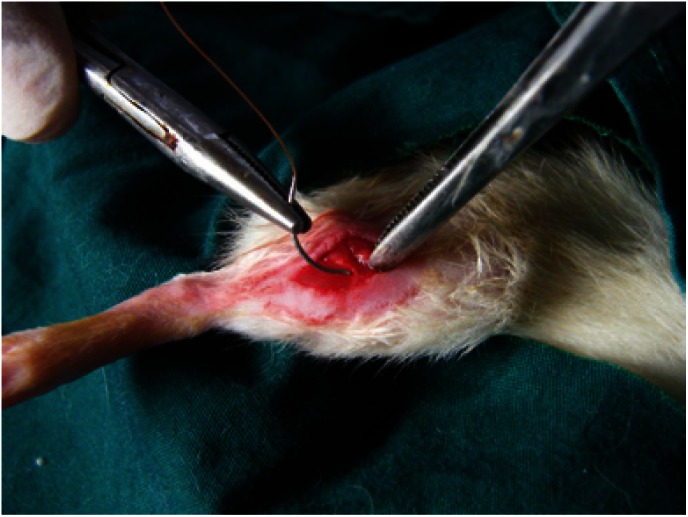
Surgical repair of the muscle using sutures after surgical exposure of the biceps femoris through a vertical incision on the lateral thigh.

**Figure 2:**
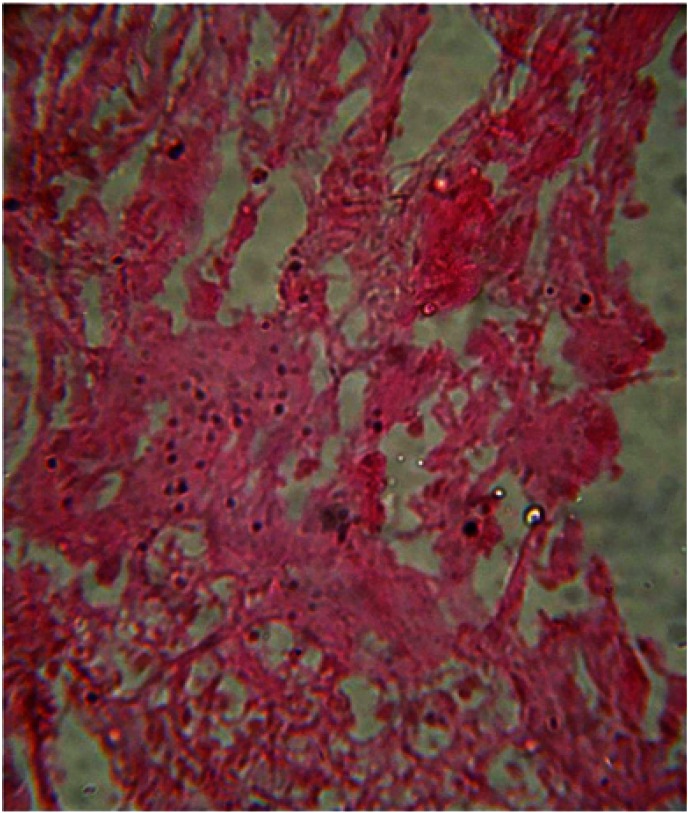
Photomicrograph at 42 hours post-surgical demonstrating minimal inflammatory component in study group-2 (PDS) (H&E staining under 40X magnification).

The skin over the lateral thigh of the animals was prepared after anaesthesia (Xylazine-Ketamine) and was disinfected with 5% Iodine solution. A vertical incision measuring 2 centimetres was made on the skin followed by blunt dissection to expose the lateral thigh muscle (biceps femoris) bilaterally (Fig. 1). A transverse incision was made on the exposed muscle, perpendicular to the direction of the muscle fibers. The muscle was repaired using the selected suture material depending on the group (Fig. 2). The skin closure was done with 3-0 nylon based on the consideration that nylon is relatively non reactive and would not have a significant effect on the healing of the underlying muscle. (4)

-Histological Evaluation

The inflammatory reaction & healing was evaluated histologically at the end of 48 hours, 1 week and 3 weeks. The sutures and the surrounding tissue were obtained for evaluation. After fixing the obtained samples in 10% formalin solution, the samples were processed for H & E staining and histopathological evaluation under a light microscope (40X magnification). The density of 4 different types of cells (Lymphocytes, Polymorphonuclear Leucocytes, Macrophages, Fibroblasts) was assessed and assigned a numerical grade from 0 to 3 using a modified Ehrlich-Hunt numerical scale (0 – Absence, 1 – Bare Scattering , 2 – Moderate , 3 – Dense Aggregation) (5). Out of the four slides prepared for each subgroup, the three most representative slides where chosen by the histopathologist for evaluation. All the tissue samples were evaluated by a single pathologist to avoid subjective variation in reporting. Each study-group gave three numerical scores. A median value was selected and standard deviation from the median was calculated for each study-group. This gave a single numerical value that was used for inter-group comparisons.

## Results

**Figure 3:**
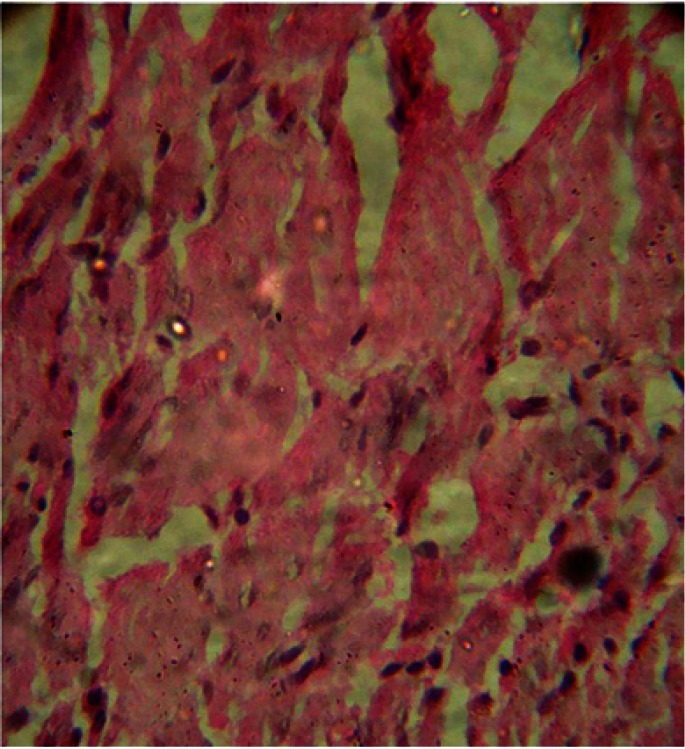
Photomicrograph at one week post-surgical showing minimal inflammatory component in study group-2 (PDS) (H&E staining under 40X magnification).

**Figure 4:**
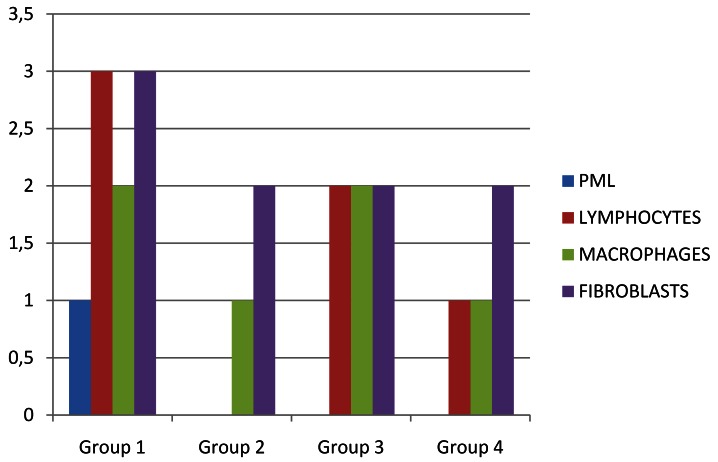
Histopathologic Scores determined in the study groups at 48 hours.

**Figure 5:**
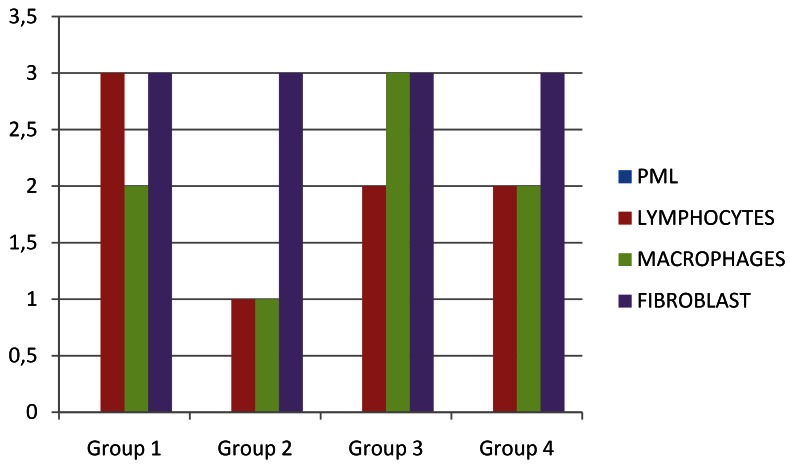
Histopathologic Scores determined in the study groups at 1 week.

On macroscopic evaluation all the surgical sites looked healthy at 48 hours, 1 week and 3 weeks postoperatively. On microscopic evaluation of the obtained biopsy specimens: At 48 hours post-operative, Group 1 samples showed severe inflammation followed by Group 3 and Group 4 in descending order of severity of inflammatory response. Group 2 with Polydiaxonone (PDS) sutures showed the least response (Fig. 3). At 1 week post surgically, catgut group had maximum amount of macrophages. Group 1 had maximum lymphocytic pro-inflammatory component at 1 week post surgery. Fibrous component was similar in all groups at 1 week (3±0). The median histopathologic scores determined in the study groups at 48 hours and 1 week are summarised in  Table 2. All tissue samples at 3 weeks had minimal or no inflammatory component that could have been assessed to evaluate the healing process. H and E staining was not found suitable to quantify the amount of fibrosis in the muscle tissue, which requires specialized staining techniques like Masson’s trichrome staining. (Figs. 4,5)

**Table 2:**
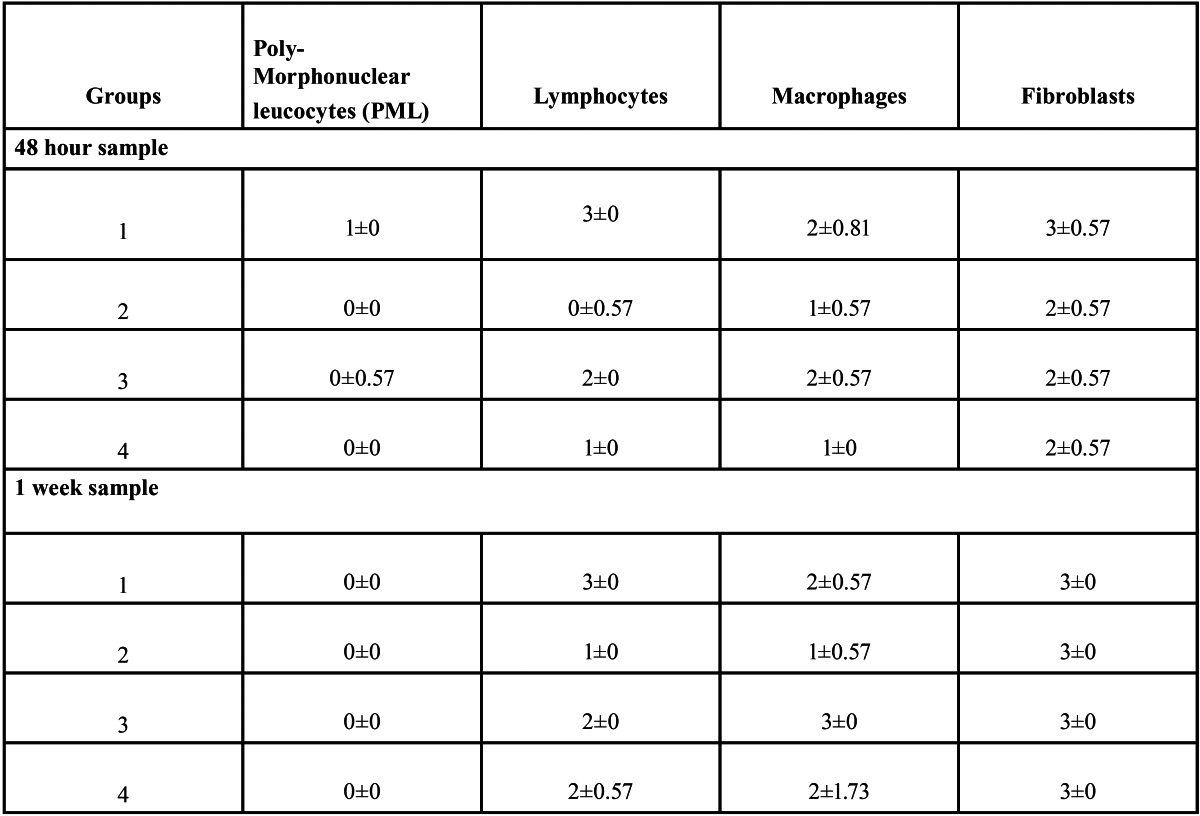
The Median Histopathologic Scores determined in the study groups at 48 hours and 1 week; Data expressed as median ±sd.

## Discussion

Surgeries in maxillofacial region warrant optimal muscle repair, because muscles in this region control two important aspects of outcome – esthetic as in facial expression and function which may include speech, deglutition and mastication. Numerous studies have been performed to assess the effects of suture materials on the skin during closure and wound approximation, while little literature is available on the response of skeletal muscle to the same. Healing of muscle tissue is different from other tissues, because of the presence of satellite cells which are myogenic precursor cells, located between the basal lamina and the plasma membrane of individual myofibres. They proliferate and differentiate into multinucleated myotubes and eventually into myofibres themselves. Interactions between inflammatory cells and skeletal muscle cells can influence muscle cell proliferation, differentiation and injury. (1,2) Tidball J.G. et al. studied the effect of macrophages on skeletal muscle healing and summarised that macrophages in particular play a beneficial role in muscle repair and regeneration through growth factors and cytokine-mediated signalling. (5)

Inflammatory reaction caused by the suture materials is one of the crucial factors that alter the healing process. It has been suggested in literature that an indirect method of assessing the healing process in a tissue sample is by studying the inflammatory process during healing. (6). In this study Nylon, Polydioxanone, Catgut and Polygalactic sutures were used due to their common usage for muscle approximation or repair. Nylon is a synthetic, nonabsorbable, monofilament suture made of a chemically inert polyamide polymer fiberand that has low tissue reactivity on skin. (6) Polydioxanone (PDS) is a synthetic, absorbable, monofilament suture made from polyester, poly (p-dioxanone) with low tissue reactivity. Polygalactic 910 is a synthetic, absorbable, braided suture made of polygalactin coated with a copolymer of L-lactide and calcium stearate. There is less of an inflammatory response due to the absorption of polygalactic acid by hydrolysis if compared with the proteolytic absorption of surgical gut. Catgut are biologic, absorbable, monofilament sutures made by twisting together strands of mostly purified collagen, in general have high tissue reactivity. (7,8)

In the present study group-1 with nylon sutures showed severe inflammation at 48 hours followed by the group with catgut suture. Polydiaxonone (PDS) sutures showed minimal inflammatory response followed by polygalactin group (Group-4) at 48 hours. Fibrous component was maximum with nylon and was moderate in all other groups at 48 hours, this may be an attempt for fibrous encapsulation. At 1 week post surgery, catgut group had maximum macrophages while the fibrous component was similar in all groups. The most probable reason of nylon sutures eliciting intense inflammatory response may be attributed to being a foreign body reaction in muscle tissue based on the finding that nylon produces more reactivity to internal tissue than external. (7) Persistence of high macrophage content at 1 week post surgical with catgut was due to the scavenging of catgut collagen fibers.

PDS sutures have obvious advantages over other sutures in surgical repair, as it is non-immunogenic as against catgut, suture retains 74% of its tensile strength after 2 weeks, 50% after 4 weeks and 25% after 6 weeks. PDS is a low reactivity suture which has been reported to maintain its integrity even in the presence of bacterial infection. The absorption rate of PDS is minimal until 90 days, and it is absorbed slowly by hydrolysis in 180 to 210 days. (8) Persistence of high macrophagic content with catgut sutures may have a beneficial effect on muscle healing, but this suture material loses its tensile strength early and muscle tissue being dynamic requires longer approximation time during the healing process.

The search for the ideal suture material depends on the minimal response elicited in the tissues added with the desirable qualities of resorption, good tensile strength and handling. In our study the most desirable suture material was determined to be PDS due to its minimal tissue response and superior handling qualities. However the fact that the presence of macrophages in healing muscle enhances the repair process would be a pointer to create an environment which contains the sustained presence of macrophages which would in turn provide a conducive atmosphere for the optimal healing of skeletal muscle in the presence of an ideal suture material. (1,5,9) The limitation of the study was the low animal sample size as approved by the ethics committee, but the study lays a foundation for evaluation of effects of sutures on healing muscle in human subjects and also skeletal muscle function after such a surgical repair using various sutures.
